# Structural Insights into Arl1-Mediated Targeting of the Arf-GEF BIG1 to the *trans*-Golgi

**DOI:** 10.1016/j.celrep.2016.06.022

**Published:** 2016-06-30

**Authors:** Antonio Galindo, Nicolas Soler, Stephen H. McLaughlin, Minmin Yu, Roger L. Williams, Sean Munro

**Affiliations:** 1 MRC Laboratory of Molecular Biology, Francis Crick Avenue, Cambridge CB2 0QH, UK

## Abstract

The GTPase Arf1 is the major regulator of vesicle traffic at both the *cis*- and *trans*-Golgi. Arf1 is activated at the *cis*-Golgi by the guanine nucleotide exchange factor (GEF) GBF1 and at the trans-Golgi by the related GEF BIG1 or its paralog, BIG2. The *trans*-Golgi-specific targeting of BIG1 and BIG2 depends on the Arf-like GTPase Arl1. We find that Arl1 binds to the dimerization and cyclophilin binding (DCB) domain in BIG1 and report a crystal structure of human Arl1 bound to this domain. Residues in the DCB domain that bind Arl1 are required for BIG1 to locate to the Golgi in vivo. DCB domain-binding residues in Arl1 have a distinct conformation from those in known Arl1-effector complexes, and this plasticity allows Arl1 to interact with different effectors of unrelated structure. The findings provide structural insight into how Arf1 GEFs, and hence active Arf1, achieve their correct subcellular distribution.

## Highlights

• Arl1 binds the N-terminal DCB domain of the Arf1-GEF BIG1
• Structure of the human DCB^BIG1^/Arl1 complex is solved at 2.3 Å resolution
• The Arl1 binding surface on the DCB domain is unrelated to known Arl1 effectors
• Structure-guided mutation shows BIG1 needs Ar11 binding for Golgi targeting

## Graphical Abstract

**Figure:**
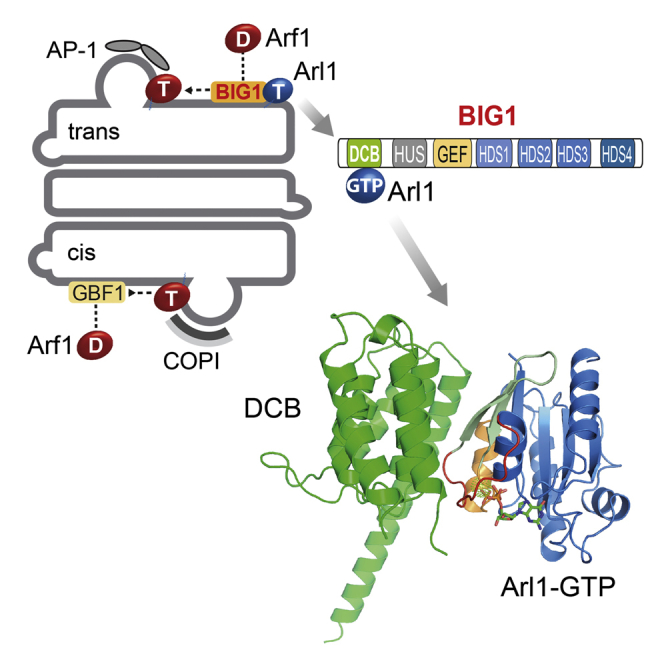

## Teaser

The GTPase Arf1 is essential for Golgi function and is activated on the *trans*-Golgi by the BIG1 exchange factor. A second GTPase, Arl1, is required for BIG1 recruitment. Galindo et al. find that Arl1 binds to the DCB domain of BIG1 and determine the structure of human Arl1 bound to this domain.

## Introduction

Membrane trafficking depends on the Arf and Rab small GTPase families (Donaldson and Jackson, 2011, Gillingham and Munro, 2007, Hutagalung and Novick, 2011). In the active (GTP-bound) state, each GTPase binds a specific set of effectors that typically includes vesicle coats, motor proteins, and vesicle tethering factors, as well as diverse regulators of organelle function. Each active GTPase is typically present on only one organelle, and so collectively, they determine the subcellular distribution of numerous proteins. Thus, understanding how GTPases are only activated in a particular location is critical to understanding the logic of sub-cellular organization. For many GTPases, it has been possible to identify specific guanine nucleotide exchange factor (GEFs) that catalyze replacement of bound GDP with GTP and so generate the active form of the GTPase. The organelle-specific targeting of these GEFs is likely to be a major factor in determining the distribution of active GTPases, but it remains poorly understood for most Rabs and Arfs, especially as almost all GEFs are themselves peripheral membrane proteins and hence will require specific interactions with GTPases and other molecules for accurate membrane targeting (Barr, 2013, Mizuno-Yamasaki et al., 2012).

Of the small GTPases that regulate membrane traffic, Arf1 has emerged as a master regulator of Golgi function (Jackson and Bouvet, 2014, Pasqualato et al., 2002). In its GTP-bound state, Arf1, and its close relatives, Arf3, Arf4, and Arf5, recruit the COPI coat to the *cis*-Golgi to generate vesicles for transport within the stack and back to the ER, while on the *trans-*Golgi, Arf1 recruits the clathrin adaptor proteins AP-1 and AP-3 to make vesicles directed to endosomes (Cherfils, 2014, Paczkowski et al., 2015). Inhibition of Arf activity by drugs or mutation causes disassembly of the Golgi apparatus and a complete block in trafficking pathways (Klausner et al., 1992, Sáenz et al., 2009).

Arf1 activation on the Golgi is mediated by two distinct families of GEFs, with mammals having GBF1 on the *cis*-Golgi, and two closely related paralogs, BIG1 and BIG2, on the *trans*-Golgi (Togawa et al., 1999, Zhao et al., 2002). Although these large soluble proteins are related over much of their ∼1,800-residue length, both GBF1 and BIG1/2 have clear orthologs in all eukaryotic kingdoms and so are thought to have diverged before the appearance of the last eukaryotic common ancestor, indicating that they have fundamentally distinct roles (Bui et al., 2009, Wright et al., 2014).

Both GBF1 and BIG1/2 contain a central Sec7 domain that catalyzes nucleotide exchange on Arf1 (Cherfils et al., 1998, Mossessova et al., 1998). However, it remains unclear how the proteins are targeted to different regions of the Golgi stack. In addition to the ∼250-residue Sec7 domain, the GBF1 and BIG1 proteins share five homology domains that are well conserved in evolution (Bui et al., 2009, Mouratou et al., 2005). Recent studies have suggested that other small GTPases bind to these domains outside of the Sec7 domain to modulate the location and extent of Arf1 activation. In particular, *Drosophila* Arf-like GTPase Arl1 binds to the N-terminal region of the fly ortholog of BIG1, and in mammalian cells, Arl1 is required for the targeting of BIG1 to the Golgi (Christis and Munro, 2012). In addition, the yeast BIG1 ortholog Sec7 binds to Arl1, and also to Arf1 itself and two Rab family proteins Ypt1 and Ypt31, and these interactions have been proposed to be involved in the Golgi recruitment and activation of Sec7 (McDonold and Fromme, 2014, Richardson et al., 2012). The interaction with Arl1 is likely to be a key determinant of the *trans*-Golgi localization of BIG1, as Arl1 specifically localizes to the *trans*-Golgi in both yeast and mammalian cells (Lu et al., 2001). Moreover, Arl1 mediates Golgi recruitment of several coil-coiled proteins that tether incoming vesicles, suggesting that Arl1 could be a master regulator of *trans*-Golgi membrane traffic (Panic et al., 2003b, Wong and Munro, 2014).

Arl1 binds to the N-terminal 559 residues of BIG1, a region that is a sufficient for Golgi targeting and that contains two predicted domains: the DCB (dimerization and cyclophilin binding) and the HUS (homology upstream of Sec7) domains (Bui et al., 2009, Mouratou et al., 2005, Christis and Munro, 2012, Mansour et al., 1999). The association of a missense mutation in the BIG2 DCB domain with a case of a familial neurodevelopmental disorder (Sheen et al., 2004) and the fact that DCB in GBF1 is needed for picornavirus replication (Belov et al., 2010) underscore the importance of the DCB domain.

To gain insight into how Arl1 regulates Arf1 activation at the *trans*-Golgi, we have mapped the Arl1 binding site on BIG1 to the N-terminal DCB domain and then determined its structure in a complex with Arl1^GTP^ at a resolution of 2.3 Å. Mutagenesis shows that interaction between the DCB domain and Arl1 is a major determinant of BIG1’s ability to recognize the *trans*-Golgi. The Arl1 binding surface on the DCB domain consists of four parallel α helices, a structure unrelated to any known Arl1 effectors, and the Arl1 residues in the binding interface show a conformational plasticity that allows it to specifically recognize structurally unrelated effectors.

## Results

### Identification of the DCB Domain as the Arl1 Binding Site

**Figure 1:**
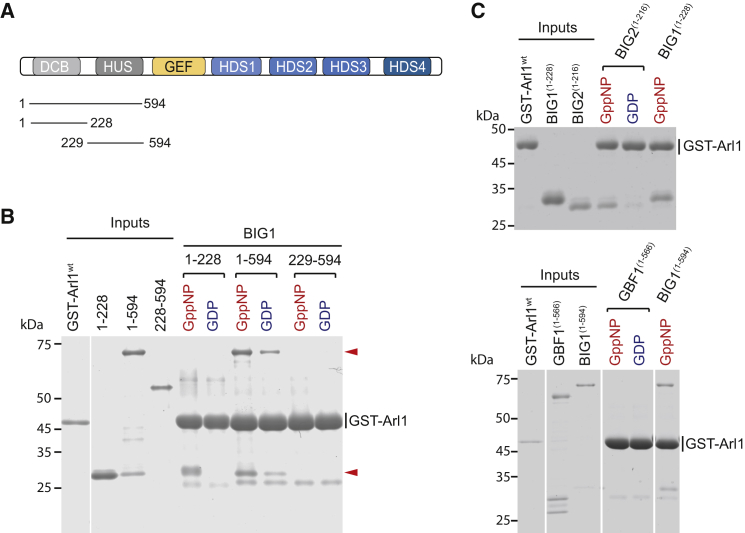
BIG1 Binds to Arl1 via the DCB Domain (A) Domain structure of the BIG family of Arf-GEFs: DCB (dimerization and cyclophilin binding), HUS (homology upstream of Sec7), and HDS (homology downstream of Sec7). (B) Coomassie-blue stained protein gel of binding assays between His_6_-tagged BIG1 fragments and GST-Arl1^ΔN14^. Input lanes contain 10% of the material used for pull-downs. Fragments containing the DCB^BIG1^ domain bound preferentially to GST-Arl1 ^ΔN14^ loaded with the GTP-analog GMP-PNP (red arrows). (C) Pull-downs assay similar to (B) but with a BIG2 N-terminal fragment (1–216) or with a GFB1 N-terminal fragment (1–566). See also Figure S1.

The N-terminal part of the *Drosophila* BIG1 homolog Sec71 binds directly to GTP-bound Arl1, and the analogous region of human BIG1 (1–559) is sufficient for Golgi targeting in mammalian cells (Christis and Munro, 2012, Mansour et al., 1999). This region contains two of the six conserved domains of the proteins, the DCB and HUS domains (Figure 1A). To map the Arl1 binding region, N-terminal fragments of BIG1 were assayed for binding to human GST-Arl1. The DCB domain (DCB^BIG1^ [1–228]) bound to Arl1^GTP^, while the HUS domain did not, with a similar result obtained with the *Drosophila* proteins (Figure 1B; data not shown). The DCB domain could also capture endogenous Arl1 from mammalian cell lysates in a GTP-dependent manner (Figure S1A).

The DCB domain was first described in GNOM, an *Arabidopsis* ortholog of GBF1 (Grebe et al., 2000). Related DCB domains in mammalian GBF1 and BIG1 were found based on sequence conservation between different species (Bui et al., 2009, Mouratou et al., 2005). The DCB domain in human BIG1 was originally annotated as residues 70–228; however, we found that while the N terminus could be truncated to residue 23 and still interact with Arl1^GTP^, larger N-terminal truncations to 51 or 61 did not bind, indicating that the actual domain is slightly larger (Figure S1B). Arl1^GTP^ shows specificity for DCB domains from proteins of the BIG family, as it also binds to the related region from BIG2, but not to the equivalent part of human GBF1 (Figure 1C).

### Crystallization of a Complex of Arl1^GTP^ Bound to the DCB Domain

To elucidate the nature of the DCB domain and the basis of its interaction with Arl1, the first 228 residues of human BIG1 (DCB^BIG1^) was coexpressed in *Escherichia coli* with human Arl1 lacking residues 1–14. These residues form an amphipathic helix that becomes fully exposed upon GTP binding, and its removal has been found to be necessary for the crystallization of GTP-bound forms of Arf-family proteins (Panic et al., 2003a, Shiba et al., 2003, Wu et al., 2004). As in previous studies, the mutation Q71L was used to ensure that the protein remained in the GTP-bound form.

**Figure 2:**
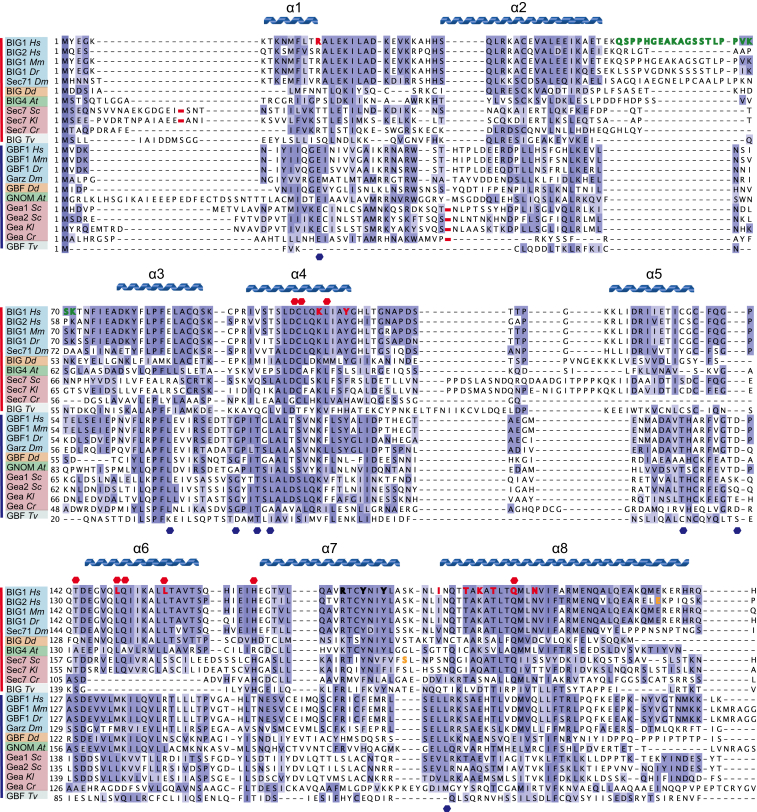
Alignment of the N-Terminal Region of the BIG and GBF Families of Arf-GEFs Alignment of sequences of BIGs and GBFs from diverse species (Hs, *Homo sapiens*; Mm, *Mus musculus*; Dr, *Danio rerio*; Dm, *Drosophila melanogaster*; Dd, *Dictyostelium discoideum*; At, Arabidopsis thaliana; Sc: Saccharomyces cerevisiae; Kl, *Kluyveromyces lactis*; Cr, Chlamydomonas reinhardtii; Tv, *Trichomonas vaginalis*). Metazoan names are blue; plants, protists, and fungi are green, orange, and gray, respectively. Conserved residues are colored according to the BLOSSUM62 matrix (threshold for visibility is 10%). Deletions (red dashes) correspond to Ser18-Gly217 in *S. cerevisiae* Sec7, Arg17-Met82 in *K. lactis* Sec7, Thr39-Asn65 in Gea1, Ser36-Asn62 in Gea2, Ser40-Asn66 in *K. lactis* Gea, and Pro36-Arg51 in *C. reinhardtii* Gea. Also indicated are conserved residues specific to the BIG family (red dot above) or to the GBF family (blue dot below). Residues 51–71 (inclusive) in human BIG1 deleted for crystallization are green. Key residues in the Arl1^GTP^/DCB^BIG1^ interaction (red bold) and the DCB-DCB interface (black bold) are shown along with the secondary structure of the DCB^BIG1^ domain. Orange bold type indicates Ser402 in *S. cerevisiae* Sec7, mutation of which to Leu results in a temperature-sensitive phenotype (Jones et al., 1999), and Glu209 in human BIG2, mutation of which to Lys causes paraventricular heterotopia (Sheen et al., 2004).

This initial complex crystallized in space group P312, with unit cell dimensions *a,b* = 96.5 Å and *c* = 201.1 Å, but the collected data were not of sufficient quality to solve the structure. Inspection of alignments of BIG1 orthologs from diverse species revealed a region between residues 51 and 71 that is poorly conserved and predicted to be disordered (Figure 2, highlighted in green). Deletion of this putative unstructured loop did not affect the interaction between Arl1^GTP^ and DCB^BIG1^ domain (Figure S1) but improved the quality of the crystals.

**Figure 3:**
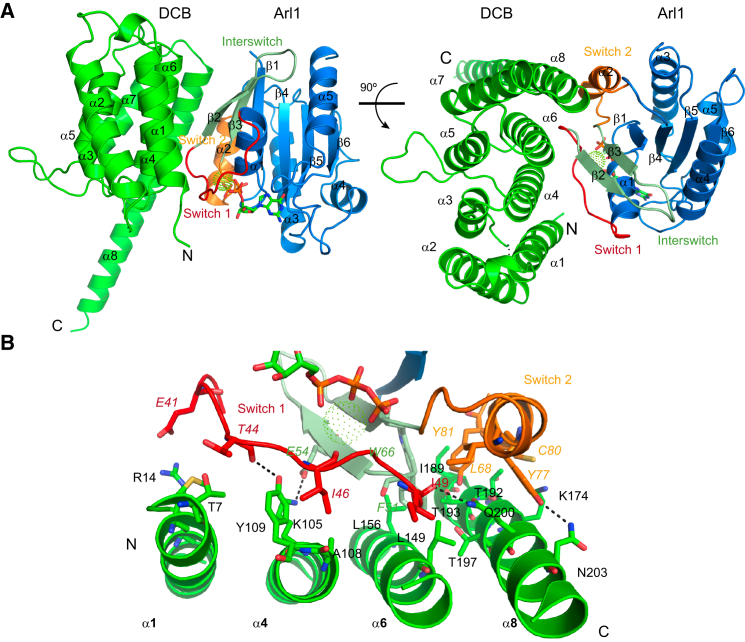
Structure of the Human Arl1^GTP^ /DCB^Δ51–71^ Complex (A) Two views of the Arl1^Q71L-GTP^/Mg^2+^/ DCB^Δ51–71^ complex. The DCB^Δ51–71^ domain (green) is formed by eight α helices and binds Arl1 via helices 1, 4, 6, and 8. Arl1 is blue, with switch 1 in red, the interswitch in pale green, and switch 2 in orange. The GTP molecule is shown as sticks, and Mg^2+^ as a sphere of green dots. (B) The Arl1-DCB domain interface. DCB α1 and α4 abut switch 1 and the interswitch of Arl1, while α6 and α8 abut the interswitch and switch 2. Side chains of residues involved in the interaction are shown as sticks. The DCB domain residues are green with black labels. The Arl1 switch 1, interswitch, and switch 2 residues are colored as in (A). Residue contacts based on hydrogen bonds with atomic distance below 3Å are indicated with black dashed lines. See also Figures S2 and S3 and Table S1.

This modified complex Arl1^Q71L-GTP^/DCB^Δ51–71^ crystallized in space group C2, with unit cell dimensions *a* = 84.2 Å, *b* = 50.7 Å, and *c* = 103.8 Å, with one complex per asymmetric unit. The X-ray structure was determined at 2.3 Å resolution (Table S1) and solved by molecular replacement using Arl1 from the Arl1^GTP^:GRIP domain complex and the N-terminal HEAT (Huntingtin, elongation factor 3, protein phosphatase 2A, and TOR1)-like repeat domain of the *Drosophila melanogaster* microtubule regulatory protein MAST/Orbit that was predicted to be distantly related to the DCB domain by HHpred (De la Mora-Rey et al., 2013, Hildebrand et al., 2009, Panic et al., 2003a). The final model includes all of the residues of the DCB^Δ51–71^ domain with the exception of three residues (71–73) adjacent to the region that was deleted (Figures 3A and S2).

### Overall Structure of Arl1^Q71L-GTP^/DCB^Δ51–71^ Domain Complex

The DCB domain is comprised of eight antiparallel α helices arranged a twisted array where helices 1, 4, and 6 are packed against helices 2, 3, 5, and 7. Helices 3–8 form a right-handed helical solenoid similar to a HEAT repeat domain (Figure 3A). The beginning of α helix 8 is also facing helix 7, but its C-terminal half protrudes out of the rest of structure. The arrangement of the helices creates two different surfaces. One formed by helices 1, 4, 6, and 8 is slightly convex and interacts with Arl1^Q71L-GTP^, and the other formed by helices 2, 3, 5, and 7 is slightly concave. The region removed to aid crystallization (51–71) connects helices 2 and 3, and the remaining link between these two α helices is not fully ordered in our model (residues 72 and 73). As expected, the Arl1^Q71L-GTP^ exhibits the typical fold of small GTPases, containing a six-stranded β sheet surrounded by five α helices (Hanzal-Bayer et al., 2002).

### The Interaction Interface between Arl1 and the DCB Domain

The interface between Arl1^GTP^ and DCB^Δ51–71^ has a total surface area of 1,509 Å^2^. Arl1^GTP^ engages with the DCB domain mainly through switch 1 and switch 2, with some additional residues from the interswitch. This accounts for the GTP-dependency of binding as these regions of Arf family G proteins undergo large conformational changes upon GTP-binding (Pasqualato et al., 2002). DCB domain α helices 1 and 4 form most of the switch 1 interactions and α helices 6 and 8 interact mainly with switch 2 (Figure 3B).

The surface on Arl1^GTP^ that interacts with the DCB domain is composed mainly of hydrophobic amino acids (Glu41, Thr44, Ile46, Ile49, Phe51, Glu54, Trp66, Tyr77, Cys80, and Asn84). Of these, those from switch 1 and the interswitch are present in many Arf family members with, for instance, all being in conserved in Arf1 (Figure S3). However, from switch 2, Tyr77 is present in only a few other Arls, and Cys80 is unique to Arl1 (Pasqualato et al., 2002). The region of the DCB domain that interacts with Arl1^GTP^ comprises twenty residues that are within 3.7 Å of the surface of Arl1. The closest, ie those within 3.3 Å or less, are the hydrophobic residues Ala108, Leu149, Leu156, Ile189, and the polar and charged residues Arg14, Lys105, Tyr109, Thr193, Lys195, Thr197, Gln200, and Asn203 (Figure 3B). Most of these residues are conserved in DCB domains across species (Figures S3A and S3B).

Comparison of GBF1 and BIG1 orthologs reveals four conserved regions: the first two map to α helices 1 and 2 in the DCB^BIG1^ structure, the third one is formed by α helices 3 and 4, and the last one includes the helices 6, 7 and 8. Some of these conserved residues are shared among BIG and GBF families, such as Lys105, Leu155, and Ala194 while others are specific to one of the two classes, such as Cys102, Leu156, and Gln200 in BIG1 (Figure 2). Close inspection of the structure shows that one of these BIG-specific residues, Gln200, makes extensive hydrophobic interactions with residue Tyr77 in switch 2 of Arl1, a residue that is involved in binding other Arl1 effectors, as discussed below.

### Structure-Based Mutational Analysis of Golgi Membrane Recruitment

**Figure 4:**
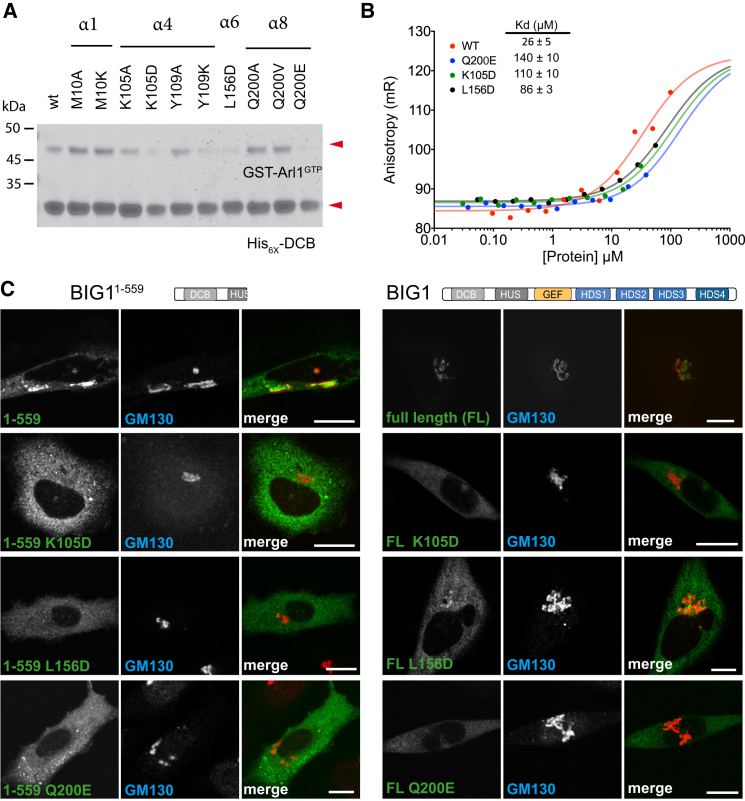
Mutational Analysis of Residues in the Arl1 Binding Interface (A) Binding assay to determine the effect of DCB mutations on binding to Arl1. The indicated versions of His_6_-DCB^BIG1^ were mixed with GMP-PNP loaded GST-Arl1^ΔN14^, and material bound to Ni-NTA beads was analyzed by SDS-PAGE and Coomassie staining. (B) Determination of the dissociation constant of the Arl1^GTP/^DCB^BIG1^ complex by fluorescence anisotropy. NT-495-labeled Arl1^GTP^ was mixed with wild-type or DCB^BIG1^ mutants. Curves fitted to a 1:1 binding model are shown, along with the calculated K_d_. (C) Confocal micrographs of HeLa cells expressing hemagglutinin (HA)-tagged forms of BIG1^1–559^ or full-length BIG1. After fixation, cells were stained for the H -tag and the Golgi marker GM130. Scale bars, 15 μm. See also Figure S4.

Next, we investigated whether the residues that form the interaction surface between Arl1^GTP^ and the DCB domain are actually important for BIG1’s ability to bind to Arl1-GTP in vitro and to localize to the Golgi in vivo. Residues located in the conserved regions of DCB α helices 4, 6, and 8 were found to be needed for binding in vitro. Thus, Lys105Asp or Tyr109Lys substitutions in Arl1-binding residues in α helix 4 prevented copurification of recombinant DCB^BIG1^ with Arl1^GTP^ (Figure 4A). Similar results were obtained when Leu156 (α helix 6) or Gln200 (α helix 8) were replaced by Asp or Glu, respectively. These mutations did not affect the folding and thermal stability of the DCB domain (Figure S4A). Mutation of other residues located in the three conserved helices 4, 6, and 8 caused weaker but reproducible effects on Arl1 binding (Figure S4B). Fluorescence anisotropy measurements of the Arl1^GTP^/DCB^BIG1^ complex in solution gave a K_d_ for binding of 26 ± 5 μM, and the Lys105Asp and Gln200Glu mutations increased the K_d_ by at least a factor of 5, with Leu156Asp having a somewhat smaller effect (Figures 4B, S4B, and S4C).

We next investigated the effect of these mutations on the ability of BIG1 to bind to the Golgi in vivo. The mutations Lys105Asp, Leu156Asp, and Gln200Glu that all had a strong effect on binding in vitro also caused both full-length BIG1 and BIG1^1–559^ to relocalize to the cytoplasm (Figure 4C). Protein blots of cell lysates confirmed that the mislocalization of the mutants is not due to protein degradation, and thus the DCB-Arl1 interaction is a major determinant of BIG1 recruitment to the Golgi (Figure S4D).

### DCB Domain Dimerization Interface

**Figure 5:**
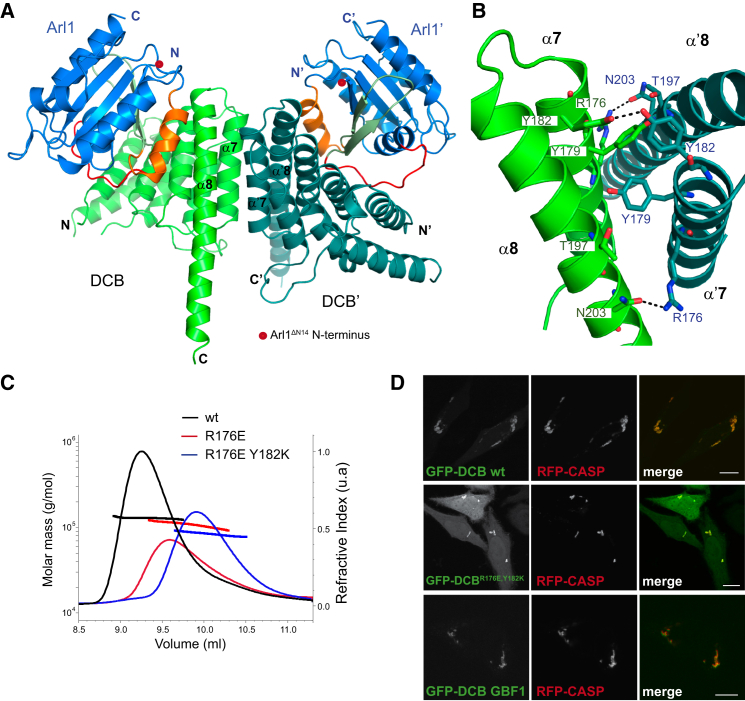
The DCB^BIG1^ Domain Dimer (A) Ribbon diagrams of two adjacent asymmetric units of the Arl1^GTP^/DCB^Δ51–71^ complex. The two DCB domains (green and turquoise) interact through helices α7 and α8. Each DCB domain binds to a molecule of Arl1 (blue, with switch 1 in red, interswitch in pale green, and switch 2 in orange) such that the two Arl1 N termini (red dots) are the same side of the complex. (B) DCB^Δ51–71^ dimer interface. Residues involved in dimerization are shown as sticks and contacts based on hydrogen bonds with a distance below 3 Å (dashed lines). (C) Determination of native molecular weight of MBP-tagged DCB^BIG1^ proteins by SEC-MALS. The expected size for a MBP-DCB^BIG1^ monomer is 69 kDa, while the average mass for the wild-type protein is 129 kDa, R176E mutant is 110 kDa, R176E/Y182K is 83 kDa. (D) Confocal micrographs of live HeLa cells expressing the N-terminal GFP-tagged DCB domains from BIG1 or GBF1 as indicated. An RFP-tagged form of the golgin CASP was used as a Golgi marker (Gillingham et al., 2002). The R176E/Y182K mutant is expressed at slightly higher levels than the wild-type protein for reasons that are unclear. It is still targeted to the Golgi, and at comparable expression levels, the mutant appeared indistinguishable from wild-type, but we show representative cells where the expression levels, and hence cytoplasmic staining, are higher. Scale bars, 5 μm.

The original description of the DCB domain reported that the related region of the plant GBF1 ortholog, GNOM, could dimerize in a yeast two-hybrid assay (Grebe et al., 2000). In addition a yeast two-hybrid screen revealed cyclophilin Cyp5 as a putative interaction partner, although Cyp5 was found to have a leader peptide and subsequently shown to be in the ER lumen, making it very unlikely that the two proteins interact in vivo (Anders et al., 2008). However, the DCB domains of human BIG1 and BIG2 were also found to form dimers, as determined by both gel filtration and analytical ultracentrifugation (Ramaen et al., 2007). In the crystal two Arl1-GTP/DCB^Δ51–71^ complexes related by two-fold crystallographic symmetry have an extensive interface (866.9 Å^2^) and suggest a dimer of heterodimers (Figure 5A). The dimerization interface comprises helices α7 and α8 that are rich in hydrophobic and basic residues. The hydroxyl group of Tyr182 is hydrogen bonded to the hydroxyl group of Tyr182 in the adjacent symmetric chain, while Arg176 is hydrogen bonded to Asn203 in the symmetric molecule (Figure 5B). The arrangement of the Arl1 proteins in the dimer would allow their N-terminal amphipathic helices to interact simultaneously with the lipid bilayer, an arrangement also seen in the two other Arl1 effectors whose structures have been solved (Nakamura et al., 2012, Panic et al., 2003a, Wu et al., 2004). However, although mutation of Arg176 and Tyr182 disrupted dimerization of the isolated domain in solution, this did not affect Golgi targeting in vivo, and so the physiological significance of this dimer is unclear (Figures 5C and 5D). Interestingly, a recent study of human GBF1 reported that mutations that disrupt dimerization in vitro had no effect on localization or activity (Bhatt et al., 2016). It may be that if the dimer exists in vivo it represents an inactive or non-membrane-bound form of the GEF.

### Interaction between Arl1 and DCB Differs from Other Arl1 Effectors

**Figure 6:**
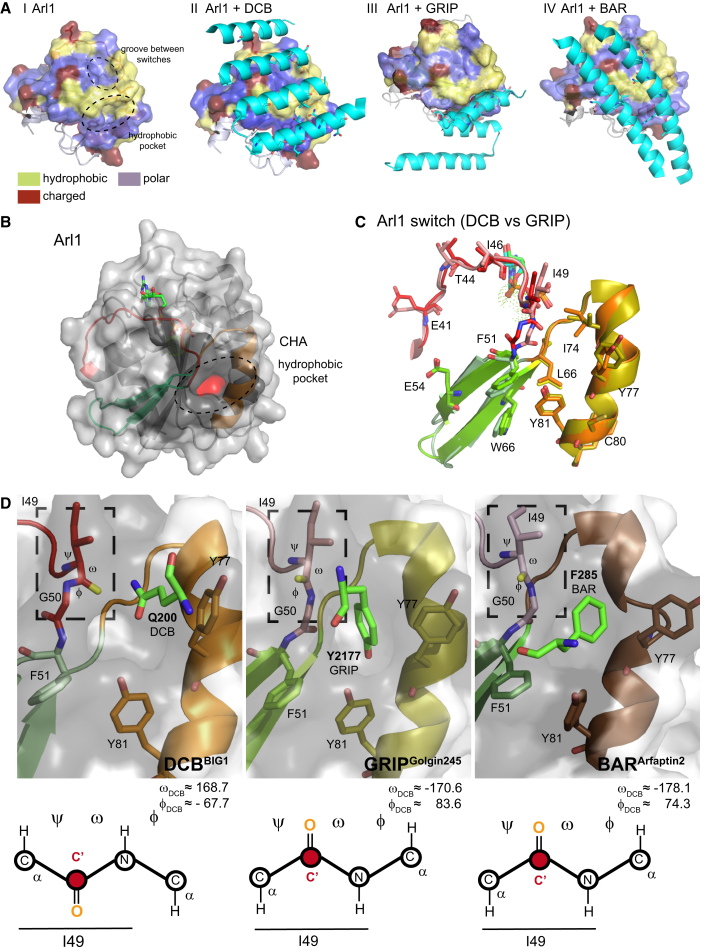
Comparison of Arl1 Effector Complexes (A) From left to right: surface representation of Arl1^GTP^ from the Arl1^GTP^/DCB^Δ51–71^ structure (I). Hydrophobic residues are yellow, polar residues purple, and charged residues brown. Ellipses indicate the hydrophobic pocket and the groove between switch 1 and switch 2. Ribbon diagram of α helices 1, 4, 6, and 8 of DCB^Δ51–71^ domain bound to Arl1^GTP^ colored as in I (II). Side chains involved in the interaction are shown as sticks. Ribbon diagram of the entire GRIP domain from golgin-245 bound to the Arl1^GTP^ colored as in I (PDB: 1UPT) (III). Ribbon diagram of α helices 2 and 3 from BAR^Arfaptin2^ bound to the Arl1^GTP^ colored as in (A) (PDB: 4DCN) (IV). (B) Arl1^GTP^ ribbon diagram/surface representation. Switch 1 is red, interswitch is pale green, and switch 2 is orange. The dark gray part of the surface indicates the common hydrophobic area (CHA), with the hydrophobic pocket in red. The GTP molecule is shown as sticks. (C) Alignment of the Arl1 switch regions from the Arl1^GTP^/DCB ^Δ51–71^ and Arl1^GTP^/GRIP^Golgin245^ complexes based on Cα position (root-mean-square deviation of 0.538 Å). Switch 1 is red or pink, the interswitch celadon or grass green, and switch 2 orange or yellow for the Arl1^GTP^/DCB^BIG1^ and Arl1^GTP^/GRIP^Golgin245^ structures, respectively. Key residues of the CHA are indicated. (D) The Arl1 hydrophobic pocket in the DCB^BIG1^, GRIP^Golgin245^, and BAR^Arfaptin2^ complexes. Arl1 residues I49, G50, F51, Y77, and Y81 are shown as sticks. Also shown is a single residue from each of the three different Arl1 effectors that projects into the pocket (bright green). Values of dihedral angles for Ile49 are indicated (rectangle). Angles are similar in the three structures, but their orientation in the DCB structure is reversed, resulting in a different position of the I49 carbonyl (C′) group, as indicated below each view. See also Figures S5.

We next compared the structure of this complex to those of the two known complexes involving other Arl1 effectors. These are the GRIP (golgin-97, RanBP2α, Imh1p, and p230/golgin-245) domain from the C terminus of golgin-245, one of several GRIP-domain-containing coiled-coil proteins that can capture endosome-derived vesicles, and Arfaptin-2, a BAR (Bin-Amphiphysin-Rvs) domain protein that is localized to the *trans*-Golgi but whose function is unclear (Man et al., 2011, Nakamura et al., 2012, Panic et al., 2003a). The three effectors are completely unrelated in structure, but all three bind Arl1 via the hydrophobic interaction surface formed by the switch regions (Figures 6A and 6B). The GRIP^Golgin245^ domain interacts mainly with Arl1 switch 2 and the hydrophobic pocket (Figures 6C and S5), but there is another hydrophobic groove formed by switch 1 and the interswitch that makes extensive contacts with the DCB^BIG1^ and the BAR^Arfaptin2^ domains (Figures 6A, 6D, and S5). Few residues outside the switch regions in Arl1 contribute to the interactions, apart from Asn84, which interacts with Ile188 in DCB^BIG1^; and Arg19, which interacts with Ile189 in DCB^BIG1^ and with Asp220 in the α2 chain of Arfaptin-2 (Nakamura et al., 2012). The major role of the switch regions in the interactions accounts for the specificity of all three effectors for the GTP-bound form of Arl1.

Khan and Ménétrey have proposed a classification scheme to highlight properties shared by domains that bind to Arf/Rab GTPases (Khan and Ménétrey, 2013). Many of these domains adopt an “all-α-helical” conformation ranging from coiled-coil structures to α-helical bundles or aspects of both. These domains usually interact with the GTPase via two helices that pack against the switch-interswitch junction. In the case of the DCB domain, the surface of interaction is actually formed by four parallel helices, two of which interact with Arl1’s switch 2 with the other two interacting mainly with switch 1.

The ability of Arl1 to recognize three very different effectors raises the question of how it can maintain selectivity for its own effectors over those of related GTPases while at the same time being promiscuous in the interactions formed by its effector-binding interface. Selectivity can be explained by the sequences of the switch regions themselves, and in particular switch 2, which is involved in effector binding in all three complexes and has residues conserved in few, if any, other members of the Arf family.

As for the structural plasticity that underlies the binding of several distinct effectors by the same GTPase, examination of other Arf:effector complexes revealed that Arf proteins generally maintain a rigid core structure independent of their binding partners (Khan and Ménétrey, 2013). Thus, the major site of conformational variability is the interaction interface itself. At the center of the interaction surface is a common hydrophobic area (CHA) of ∼480 Å (Figure 6B) (Khan and Ménétrey, 2013, O’Neal et al., 2005). A feature of the CHA is the aromatic triad Phe51, Trp66, and Tyr81 that is highly conserved in Arfs and Rabs and found in some cases to undergo rotational isomerization to allow binding to different effectors (Khan and Ménétrey, 2013, Recacha et al., 2009). However, this does not appear to be the case for Arl,1 as these three residues vary little among the three complexes.

The other conserved feature of the CHA is the hydrophobic pocket located at the interface between switch 1, the interswitch, and switch 2, and in Arl1 comprising nine residues: Ile49 and Gly50 from switch 1; Phe51, Val53, and Trp66 from the interswitch; and Ile74, Tyr77, and Tyr81 from switch 2 (Figure 6B). Comparison of the three structures reveals that the largest differences in the interfacial side chains are in the residues in switch 1 that flank the hydrophobic pocket (Figure 6C). In the case of the GRIP domain, Tyr2177 projects into the hydrophobic pocket to hydrogen bond with Tyr81 from Arl1 (Figure 6D). Likewise, the Arfaptin-2 BAR domain also projects Phe285 inside the hydrophobic pocket. In both cases, the carbonyl group of Arl1 Ile49 is facing out of the hydrophobic pocket (Figure 6D). In contrast, in the Arl1^GTP^/DCB^Δ51–71^ complex, the carbonyl group of Arl1 Ile49 remains buried inside the rim of the hydrophobic pocket (Figure 6D). The switch between these two conformations is possible because of the adjacent Gly50 residue. For the DCB interaction, the Arl1 hydrophobic pocket is locked by Gln200 from the DCB domain, which does not protrude deeply into the pocket, but its amide group is hydrogen bonded to the oxygen from the carbonyl of Ile49, and the phenyl group of Tyr77 is rotated by 70° (relative to that in the GRIP domain complex) so as to face the side chain of Gln200 (Figure 6D). Previous studies on the GTPase Arl2 have also found two distinct orientations for the carbonyl before Gly50 in complexes with two different effectors (Chavrier and Ménétrey, 2010, Hanzal-Bayer et al., 2002, Zhang et al., 2009). Indeed, this glycine residue is conserved in all Arf family members except Sar1 and in the Rab and Ran families as well, suggesting that it is key contributor to the structural plasticity that allows these GTPases to bind to multiple effectors.

## Discussion

Our work has provided structural insight into the regions that flank the central Sec7 catalytic domain of the two major classes of Golgi Arf GEF. In addition, it shows how Arl1 interacts with BIG1 via the DCB domain. Although some of the conserved residues in the BIG1 DCB domain are specific to the BIG family, this DCB region is also present in the GBF1 family and in the more distantly related MON2 and BIG3 proteins that lack GEF activity and are of unknown function (Efe et al., 2005, Gillingham et al., 2006, Li et al., 2014). Thus, it seems possible that the DCB domain of these other proteins will also contribute to membrane recruitment by binding other small GTPases. Indeed, we found that the DCB domain of human GBF1 is sufficient for Golgi targeting (Figure 5D). This DCB domain does not bind Ar1l, consistent with GBF1 being on the *cis*-Golgi (Figure 1C). The first 380 residues of GBF1 that include the DCB domain have been reported to interact with Rab1B (Monetta et al., 2007), but we were not able to detect an interaction between GBF1 fragments DCB^(1–215)^ or DCB-HUS^(1–566)^ and either Rab1A or Rab1B (A.G., unpublished data).

Another notable feature of the DCB domain is that it has a HEAT repeat structure. Strikingly, the structural-based homology detection program HHpred (Hildebrand et al., 2009) not only detects that DCB is related to known HEAT repeat proteins but also predicts a similar relationship for large sections of the rest of the regions of BIG1 and GBF1 that flank the Sec7 domain. Thus, it seems quite possible that these proteins are elongated molecules consisting of the Sec7 domain flanked by HEAT-repeat-based solenoids that serve as flexible scaffolds for interaction with membrane landmarks and regulatory molecules (Andrade et al., 2001). Given this likely elongated structure for these large proteins, it seems improbable that Arl1 is the only protein that interacts with BIG1 (Wright et al., 2014). Indeed, studies on yeast Sec7 have shown an interaction not only with Arl1 but also with Arf1, Ypt1, and Ypt31 (McDonold and Fromme, 2014). The DCB domain of yeast Sec7 also appears important for Golgi targeting, as the yeast *sec7-1* allele, a thermosensitive mutant that carries the substitution S402L in the DCB domain (Figure 2), results in mislocalization of GFP-Sec7-1 at the restrictive temperature (Richardson et al., 2012). Sec7’s interaction with Arf1 may provide a positive feedback loop to increase recruitment, but it seems unlikely to have a role in specificity as Arf1-GTP is also abundant on the *cis*-Golgi. The interactions with Ypt1 and in particular Ypt31 appear to activate exchange activity by relieving inhibitory interactions following membrane binding (McDonold and Fromme, 2014). The relevance of these findings to mammalian cells is unclear, and indeed, in mammals, the orthologs of Ypt1 (Rab1) and Ypt31 (Rab11) are on the *cis*-Golgi and recycling endosomes, respectively. Nonetheless, although our results demonstrate that, in mammals, Ar1l is critical for bringing BIG1 to the Golgi, the rest of BIG1 could help to stabilize membrane binding, regulate GEF activity, or even scaffold interactions with other proteins once BIG1 is membrane associated.

**Figure 7:**
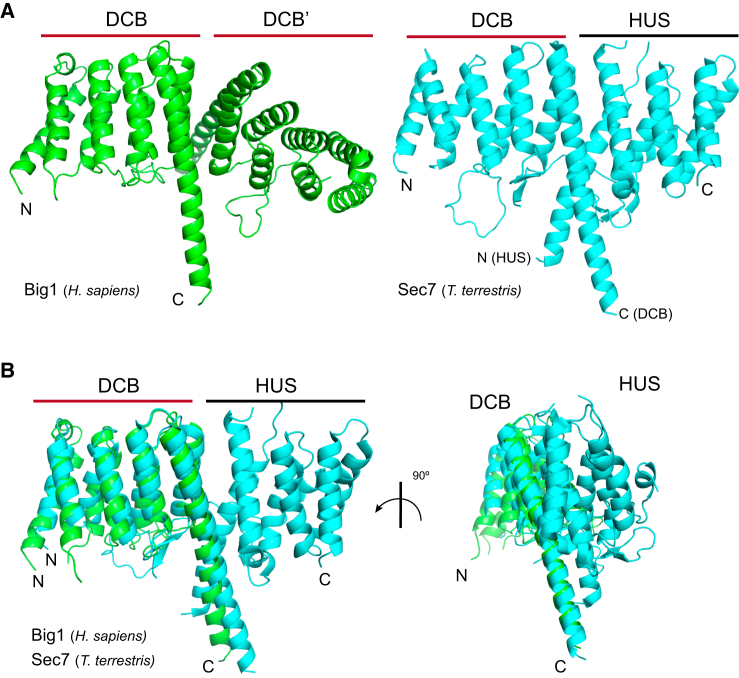
Comparison between the Structures of DCB^(Δ51–71) BIG1^ and DCB-HUS Sec7 (A) Ribbon diagrams of the DCB^Δ51–71^/DCB^Δ51–71′^ dimer from human BIG1 (green) and the structure of the DCB-HUS region from *Thielavia terrestris* Sec7 (blue, PDB: ID 5HAS). (B) Two views of an alignment of human BIG1 DCB^Δ51–71^ and the DCB domain from the DCB-HUS *T. terrestris* Sec7 structure, with coloring as in (A). The root-mean-square deviation is 8.4 Å, with this relatively high value primarily reflecting differences in the conformation of the loop between α helices 4 and 5 and in the tilt of α helix 8.

In addition to the findings described here, a recent paper has reported the structure of an N-terminal region of Sec7 from the thermophilic filamentous fungus *Thielavia terrestris* that contains both the DCB and adjacent HUS domain (Richardson et al., 2016). The overall fold of the DCB domain is the same as that we find for human BIG1, and the HUS domain is confirmed to comprise a HEAT-repeat solenoid as predicted by HHPRED (Figures 7A and 7B). Two interesting features emerge from comparing the two studies. First, although Sec7 from the budding yeast *Saccharomyces cerevisiae* is reported to bind Arl1, the DCB-HUS region of the protein does not appear to bind Arl1 (Richardson et al., 2016). However, although the 20 residues that form the Arl1 interface in human BIG1 are very well conserved in metazoans (eg 18 are identical in *Drosophila* with two conservative changes), they are not well conserved in yeast (only six are identical in *S. cerevisiae* Sec7). Thus, it appears that in yeasts Arl1 binds to a region of the protein other than the DCB domain recognized in metazoans. The second interesting feature revealed by comparing the structures is that in the DCB-HUS structure, the HUS domain covers the region on the DCB domain that forms the DCB homo-dimer interface reported here (Figure 7B). This raises the possibility that the ability of the isolated DCB domain to form a dimer in vitro could simply be a consequence of removing the HUS domain. However, it is striking that the junction between the DCB and HUS domains involves two helices that, unlike the rest, project out of the solenoid. Moreover, in most species, these helices are connected by a linker of ∼100 residues that is poorly conserved and predicted to be disordered. This raises the possibility that under some circumstances, the DCB and HUS domains separate to allow the DCB domain to form a homodimeric interaction or to even pair with the HUS domain on a second molecule as has been suggested previously (Ramaen et al., 2007). Indeed, although the *T. terrestris* DCB-HUS fragment that was crystalized is monomeric in solution, the equivalent region of *S. cerevisiae* Sec7 is dimeric (Richardson et al., 2016).

A full understanding of the organization of these Arf GEFs and how this relates to their activity will probably require the structure of a complete protein. Nonetheless, our structural and mutagenesis data show that Arl1 is a major determinant for recruiting BIG1 to the *trans*-Golgi, and they identify the DCB domain as a small GTPase binding region for at least the mammalian large Arf GEFs. These data thus open the way to a structurally informed dissection of the targeting and regulation of these key components of membrane traffic.

## Experimental Procedures

### Protein Expression and Purification

Human Arl1 (UniProt P40616, residues 15–181), carrying the Q71L mutation, and human BIG1 (UniProt Q9Y6D6) fragment DCB^BIG1^ (residues 1–228) were co-expressed from pOPTC (Panic et al., 2003a) in BL21-(DE3)-RIPL as GST-Arl1^Q71L^ and His_6_-DCB^BIG1^. The same strategy was followed for the coexpression of Arl1^Q71L^ and the BIG1 fragment DCB^Δ51–71^ (residues 1–228 with a deletion of 21 residues [51–71 inclusive]). Cells expressing either Arl1^Q71L^/DCB^BIG1^ or Arl1^Q71L^/DCB^Δ51–71^ were grown at 37°C until optical density 600 (OD_600_) ∼1 and induced with 0.2 mM IPTG at 15°C for 16 hr. Cells were pelleted, resuspended in buffer A (50 mM Tris-HCl [pH 7.5], 100 mM NaCl, 5 mM MgCl_2_, 1 mM DTT, and 0.2 mM GTP) and lysed with an Emulsiflex-C3 at 15,000 psi at 4°C. The cell lysate was cleared by centrifugation at 16,000 × *g* for 30 min at 4°C and mixed with glutathione-Sepharose beads (GE Healthcare). After three washes of ten bed volumes, resin-bound material was released with 0.08 mg TEV protease per 10 mg complex for 12 hr at 4°C. The Arl1^Q71L^/DCB complexes were further purified by gel filtration on a Superdex 75 16/60 column equilibrated in buffer B (20 mM Tris-HCl [pH 7.5], 100 mM NaCl, 1 mM MgCl_2_, 1 mM DTT, and 0.01 mM GTP). After elution, fractions were concentrated to 12–14 mg/ml and snap frozen in liquid nitrogen.

### Crystallization

Initial crystals of Arl1^Q71L^/DCB^BIG1^ and Arl1^Q71L^/ DCB^Δ51-71^ complexes were obtained by vapor diffusion in sitting drop at 18°C. Drops were made by mixing 50 nl protein solution and 50 nl reservoir solution (Arl1^Q71L^/DCB^BIG1^: 17%–22% [w/v] PEG4000, 100 mM Tris-HCl 8.5, 150 mM Li_2_SO_4_; Arl1^Q71L^/ DCB^Δ51–71^: 10% [w/v] PEG8000/PEG1000, 100 mM Tris-OAc [pH 8.5], and 0.1–0.2 mM NaOAc). Microseeding was used to increase crystal nucleation. Crystals were harvested and cryo-cooled in reservoir solution supplemented with 25% (w/v) PEG4000 for Arl1^Q71L^/DCB^BIG1^ or with 20% (v/v) glycerol in the case of Arl1^Q71L^/ DCB^Δ51–71^.

### Data Collection, Phasing, and Model Refinement

Diffraction data were collected at −100°C at beamlines I03 and I04 at the Diamond Light Source (STFC-UK). Crystallographic data were processed with XDS (Kabsch, 2010) or iMOSFLM (Powell et al., 2013) and reduced and scaled using Pointless and Aimless from the CCP4 suite (Winn et al., 2011). Crystal structures were solved by molecular replacement using Phaser (McCoy et al., 2007). Search models were Arl1^Q71L-GTP^ (PDB: 1UPT) (Panic et al., 2003a) and the N-terminal region of MAST/Orbit (PDB: 4G3A) (De la Mora-Rey et al., 2013). Model rebuilding was done with Coot (Emsley et al., 2010) and PHENIX (Adams et al., 2010). Final statistics for the 2.3-Å resolution model are given in Table S1. The accession number for the coordinates and structure-factor amplitudes of Arl1^GTP^/DCB^Δ51–71^ is PDB: 5EE5. Identification of key residues involved in Arl1^GTP^/DCB^BIG1^ interaction and DCB^BIG1^/DCB^BIG1^ were based on results obtained from NCONT and CONTACT from CCP4 (Winn et al., 2011). Figures were produced using PyMOL 1.7 and surface conservation assessed with ConSurf (Landau et al., 2005).

### Fluorescence Anisotropy Experiments

The binding of DCB variants to Arl1, labeled with NT-495 (NanoTemper Technologies), was measured by fluorescence anisotropy at 20°C using a PHERAstar plate-reader (BMG Labtech). The reaction was followed in 50 mM Tris-HCl (pH 8.0), 110 mM KCl, 1 mM DTT, 5 mM MgCl_2_, 0.001% (v/v) Triton X-100, 0.1% (v/v) Tween-20, and 5 μM GTP. A control reaction with free NT-495 label showed no change in anisotropy with increasing protein concentration. Using a single-site binding model, the data were fitted to the equation:

$$F=F0\;+\;\frac{\left(F1-F0\right)\;\left\{\left(\left[PT\right]+\left[LT\right]+Kd\right)-\sqrt{\left(\left[PT\right]+\left[LT\right]+KD\right)2-4\left[PT\right]\left[LT\right]}\right\}}{2\left[PT\right]},$$

where F_0_ and F_1_ are the anisotropy in the absence of titrating protein and at saturation respectively, [LT] and [PT] are the total concentrations of DCB^BIG1^ and labeled Arl1^GTP^, and *K*_*d*_ is the dissociation constant. For all the mutant DCB proteins, except K105A/Q201V, Q200E, and L156D, the value of *F1* was fixed at the value fitted for the wild-type.

### Circular Dichroism

Circular dichroism (CD) scans were recorded between 190 and 260 nm using a JASCO-815 CD spectrophotometer at a protein concentration of 8 μM in PBS at 20°C. Control spectra of buffer were used to subtract the baseline contribution to the signals. Thermal denaturations were preformed over the range of 4°C to 95°C at a rate of 1°C/min. Data were fitted to a Boltzmann curve with sloping baselines:

$$Yobs=\left(Y0N+\alpha NT\right)+\;\left[\frac{\left(Y0N\;+\;\beta DT\right)-\left(Y0N+\alpha NT\right)}{1+\exp\left\{\frac{\left(Tm-T\right)}{C}\right\}}\right],$$

where Y_obs_ is the observed CD signal at temperature T; Y_N_ and Y_D_ are the native and denatured protein CD contributions with α_N_ and β_D_ are the slopes for the native and denatured signals, respectively; T_m_ is the melting temperature; and C is a constant. To compare protein stability of DCB^BIG1^ variants, CD thermal denaturation data were transformed using the fitted baselines to the CD scans data, and they were plotted as fraction of unfolded protein (f_D_) versus temperature:

$$\int D=\;\frac{Yobs-\left(Y0N+\alpha NT\right)}{\left(Y0D+\;\beta DT\right)-\left(Y0N+\;\alpha NT\right)}.$$

### SEC-MALS Analysis

MBP-tagged DCB^BIG1^ proteins were resolved on a Superdex-75 HR10/300 analytical gel filtration column (GE Healthcare) at 0.5 ml/min in PBS (pH 7.5), 150 mM NaCl, 5 mM MgCl_2_, and 0.001% (w/v) Triton X-100 before detection on a Wyatt Heleos II 18 angle light-scattering instrument coupled to a Wyatt Optilab rEX online refractive index detector and determination of native molecular weight (Perica et al., 2014).

### Binding Assays

GST-Arl1 (residues 15–181) and His_6_-tagged N-terminal fragments of human BIG1, BIG2 (UniProt Q9Y6D5) and GBF1 (UniProt Q92538) were expressed in BL21-CodonPlus (DE3)-RIPL cells and lysates prepared as described above. His_6_-tagged proteins were purified using Ni-NTA beads (QIAGEN) and GST-Arl1 using glutathione-Sepharose (GE Healthcare). Purified protein were eluted and desalted in buffer C (20 mM Tris-HCl, 110 mM KCl, 0.1 mM Mg_2_Cl, 1 mM DTT, and 0.1% Triton X-100). Purified GST-Arl1 was loaded with nucleotide by incubation at 37°C for 30 min in 10 mM EDTA and either 200 μM GMP-PNP or 1 mM GDP and then by increasing the MgCl_2_ to 20 mM and cooling to 4°C. 2 nM GST-Arl1^GMP-PNP^ or GST-Arl1^GDP^ was mixed with 2 nM of the His_6_-tagged protein in 500 μl buffer C plus 5 mM MgCl_2_ and rotated at 4°C for 30 min before adding 10 μl 50% glutathione-Sepharose beads. Beads were washed thrice with 1 ml buffer C plus 5 mM MgCl_2_ and bound material eluted with SDS buffer and analyzed by SDS-PAGE. 10 μl Ni-NTA beads preloaded with His_6_-tagged DCB^BIG1^ variants was mixed with 1 nM GST-Arl1^GMP-PNP^ in 500 μl buffer C plus 5 mM Mg_2_Cl and 40 mM imidazole. Beads were washed thrice with 1 ml supplemented buffer C and bound material eluted with 20 μl of SDS buffer and analyzed by SDS-PAGE.

### Microscopic Imaging

HeLa Cells were transfected using FuGene 6 (Promega) and plated onto multispot microscope slides 12 hr after transfection. Cell fixation, permeabilization, staining, and confocal imaging (Zeiss LSM 780) were performed as described previously (Wong and Munro, 2014). Live cell images were acquired at 37°C using a stage incubator and four-well chambers (Nunc Lab-Tek) in media supplemented with fetal bovine serum and 20 mM HEPES (pH 7.2).

## Author Contributions

A.G. conceived and performed experiments and wrote the manuscript, S.M. conceived the study and experiments and wrote the manuscript, N.S. did the phasing and model refinement, M.Y. contributed to data acquisition, and S.H.M. and R.L.W. provided expertise and feedback.

## Accession Numbers

The accession number for the *H. sapiens* Arl1^Q71L^/ DCB^Δ51–71^ complex reported in this paper is PDB: 5EE5.

## Supplemental Information

Document S1. Supplemental Experimental Procedures, Figures S1–S5, and Tables S1

Document S2. Article plus Supplemental Information
