# Supplementary material for: Structural Insights into Arl1-Mediated Targeting of the Arf-GEF BIG1 to the trans-Golgi
Source: Cell Rep. 2016 Jun 30;16(3):839–50. doi: 10.1016/j.celrep.2016.06.022 (PMC4956616; doi:10.1016/j.celrep.2016.06.022)
Supplement: Document S1. Supplemental Experimental Procedures, Figures S1–S5, and Tables S1 [file mmc1.pdf]

Cell Reports, Volume *16*

Supplemental Information

Structural Insights into Arl1-Mediated Targeting of the Arf-GEF BIG1 to the *trans*-Golgi

Antonio Galindo, Nicolas Soler, Stephen H. McLaughlin, Minmin Yu, Roger L. Williams, and Sean Munro

Figure S1

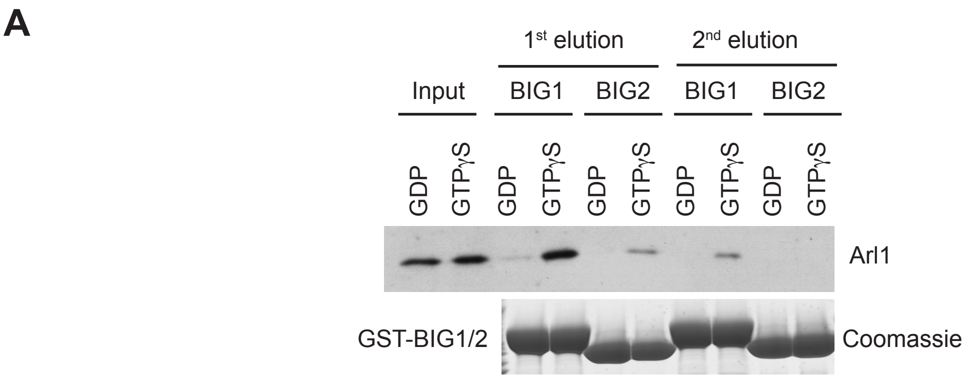
A
1st elution
2nd elution
Input
BIG1
BIG2
BIG1
BIG2
GDP
GTPγS
GDP
GTPγS
GDP
GTPγS
GDP
GTPγS
GDP
GTPγS
Arl1
GST-BIG1/2
Coomassie

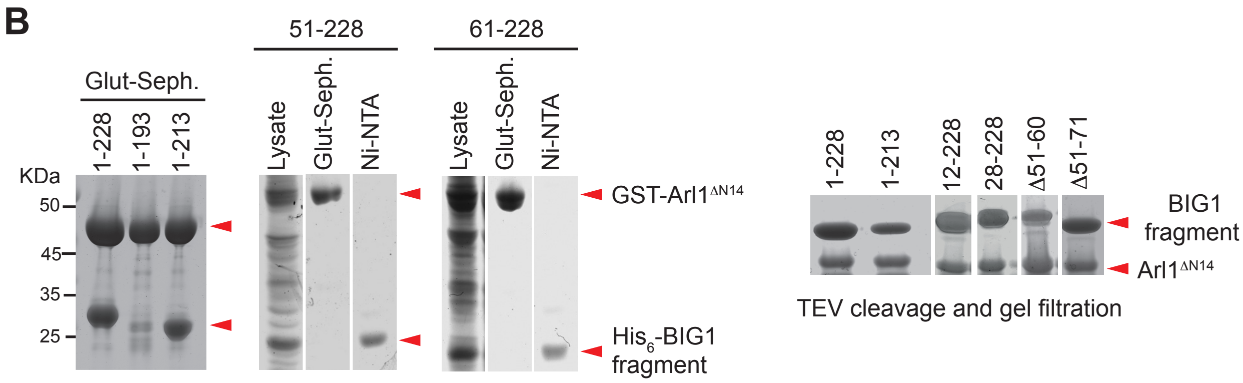
B
Glut-Seph.
1-228
1-193
1-213
KDa
50
45
35
25
51-228
Lysate
Glut-Seph.
Ni-NTA
61-228
Lysate
Glut-Seph.
Ni-NTA
GST-Arl1ΔN14
His6-BIG1
fragment
1-228
1-213
12-228
28-228
Δ51-60
Δ51-71
BIG1
fragment
Arl1ΔN14
TEV cleavage and gel filtration

Figure S2

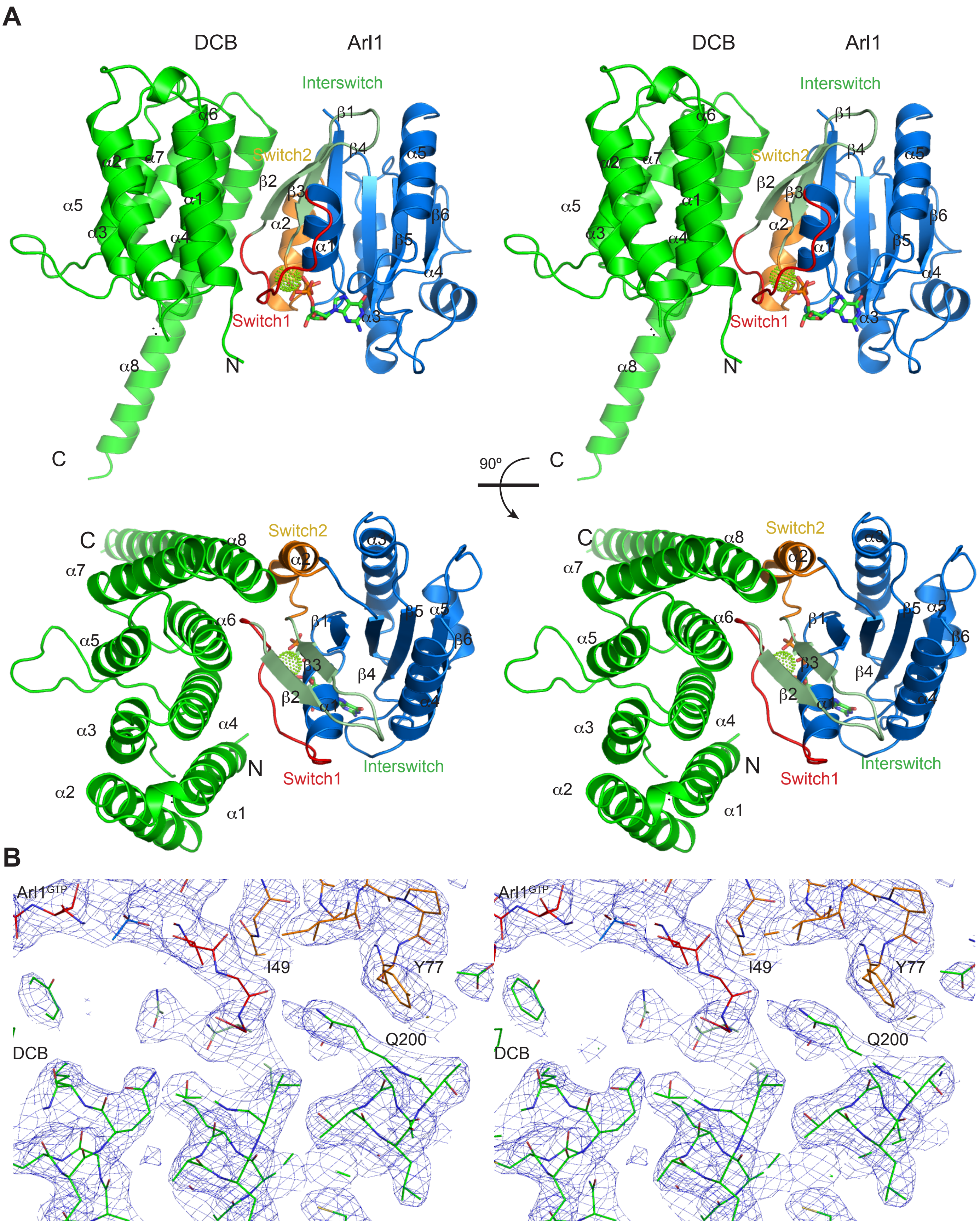
A
DCB
Arl1
Interswitch
Switch2
Switch1
N
C
90º
B
Arl1GTP
I49
Y77
Q200
DCB

Figure S3

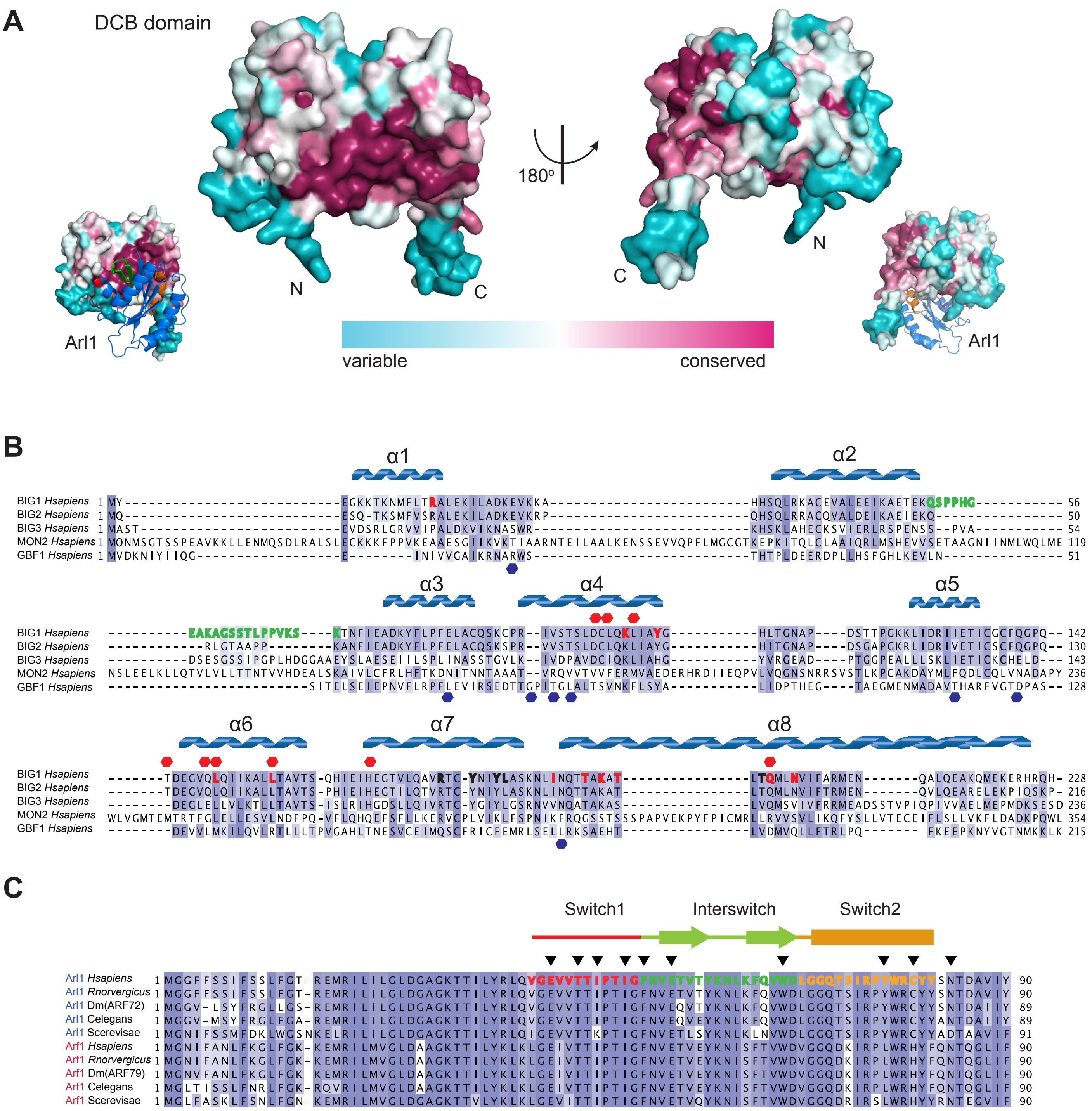
A
DCB domain
Arl1
N
C
180°
variable
conserved
Arl1
B
α1
α2
α3
α4
α5
α6
α7
α8
BIG1 Hsapiens
BIG2 Hsapiens
BIG3 Hsapiens
MON2 Hsapiens
GBF1 Hsapiens
C
Switch1
Interswitch
Switch2
Arl1 Hsapiens
Arl1 Rnorvergicus
Arl1 Dm(ARF72)
Arl1 Celegans
Arl1 Scerevisae
Arf1 Hsapiens
Arf1 Rnorvergicus
Arf1 Dm(ARF79)
Arf1 Celegans
Arf1 Scerevisae

Figure S4

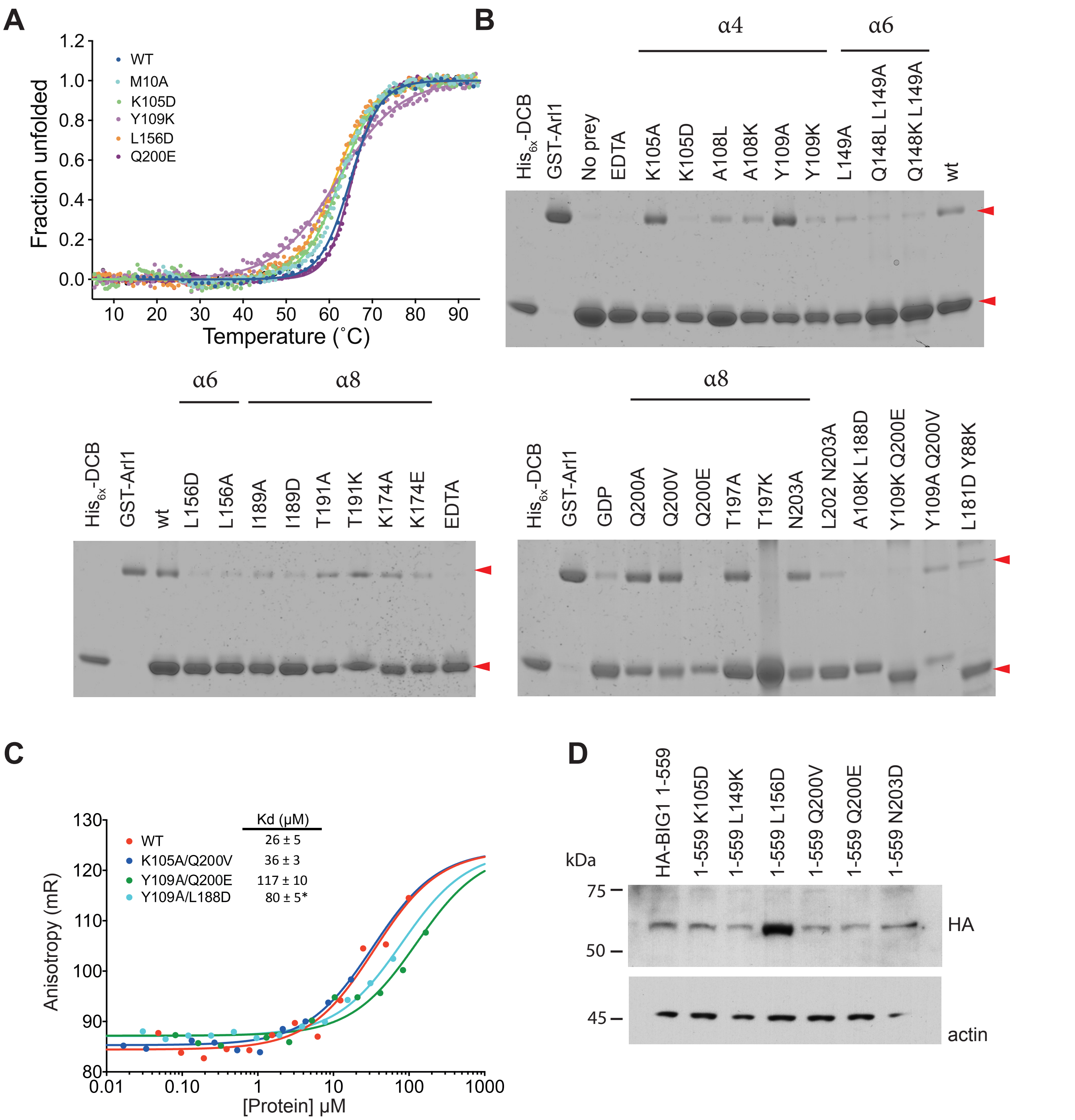

A
Fraction unfolded
1.2
1.0
0.8
0.6
0.4
0.2
0.0
10 20 30 40 50 60 70 80 90
Temperature (˚C)
WT
M10A
K105D
Y109K
L156D
Q200E
B
α4
α6
His6x-DCB
GST-Arl1
No prey
EDTA
K105A
K105D
A108L
A108K
Y109A
Y109K
L149A
Q148L L149A
Q148K L149A
wt
α6
α8
His6x-DCB
GST-Arl1
wt
L156D
L156A
I189A
I189D
T191A
T191K
K174A
K174E
EDTA
α8
His6x-DCB
GST-Arl1
GDP
Q200A
Q200V
Q200E
T197A
T197K
N203A
L202 N203A
A108K L188D
Y109K Q200E
Y109A Q200V
L181D Y88K
C
Anisotropy (mR)
130
120
110
100
90
80
0.01 0.10 1 10 100 1000
[Protein] μM
Kd (μM)
WT 26 ± 5
K105A/Q200V 36 ± 3
Y109A/Q200E 117 ± 10
Y109A/L188D 80 ± 5*
D
HA-BIG1 1-559
1-559 K105D
1-559 L149K
1-559 L156D
1-559 Q200V
1-559 Q200E
1-559 N203D
kDa
75
50
45
HA
actin

Figure S5

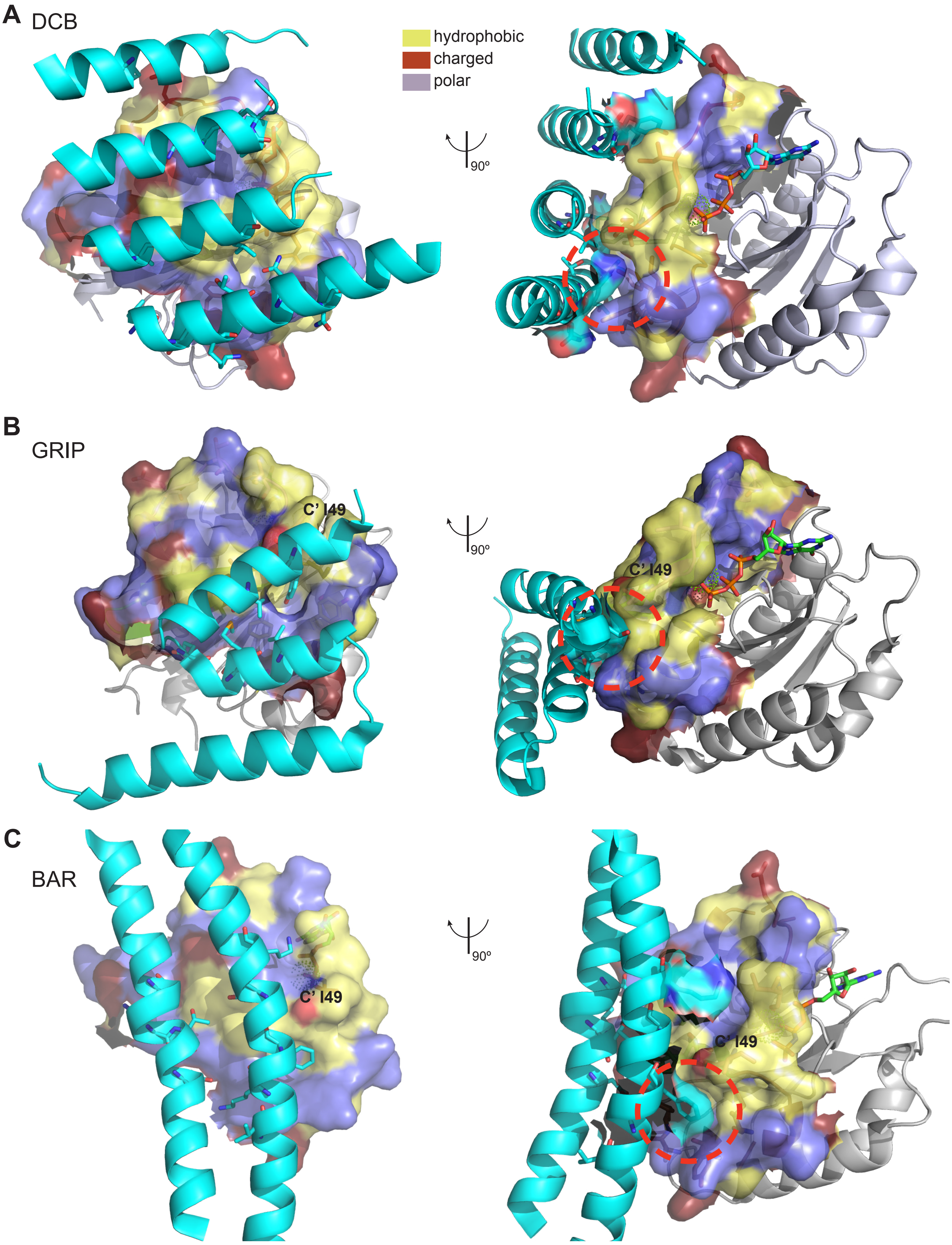
A
DCB
hydrophobic
charged
polar
90º
B
GRIP
C' I49
90º
C' I49
C
BAR
C' I49
90º
C' I49

Figure S1. Mapping of the region of BIG1 that binds to Arl1, Related to Figure 1.

(A) Arl1 from GTPγS-containing cell lysates binds to the DCB domain from BIG1 and BIG2. HeLa cell lysates were supplemented with 10 mM EDTA and 0.2 mM GTPγS or GDP and incubated at 30°C for 20 minutes. After addition of 60 mM $MgCl_2$ the lysates were incubated at 4°C with glutathione-Sepharose beads coated with GST-DCB^{BIG1} or GST-DCB^{BIG2}. Bound material was eluted with 2 M NaCl and immuno-blotted for Arl1.

(B) Coomassie-stained gels of GST-Arl1^{GTP}/BIG1$^{N\text{-terminal}}$ complexes purified by affinity chromatography and, where indicated, TEV cleavage and gel filtration. A fragment comprising residues 1-228 bound to Arl1^{GTP}, as did a C-terminal truncation to residue 213, but not to residue 193. N-terminal truncations to residues 12 or 28 could still bind, but removal of the first 51 or 61 residues prevented binding. Removal of a disordered loop between residues 51 and 71 (inclusive) from the 1-228 fragment did not prevent the formation of the complex.

Figure S2. The human Arl1^{GTP}/DCB$^{\Delta51\text{-}71}$ complex in stereo, Related to Figure 3.

(A) Stereo views of a ribbon diagram of the Arl1$^{Q71L\text{-}GTP}$/Mg^{+2}/ DCB$^{\Delta51\text{-}71}$ complex. Views are rotated 90º degrees with respect to each other. The DCB$^{\Delta51\text{-}71}$ domain is colored in green. Arl1 is shown in blue, with switch 1 colored red, interswitch in pale green and switch 2 in orange. The GTP molecule is shown as sticks with the Mg^{2+} ion as a sphere of green dots.

(B) Stereo view of a representative portion of the $2F_o\text{-}F_c$ electron density map (contoured at 2.0 σ). The map is centered on the Arl1^{GTP}-DCB^{BIG1} interface. The modeled structure is shown as sticks and colored as in (A). Also labeled are the residues DCB^{BIG1} Gln200 and Arl1 Ile49 and Tyr77.

Figure S3. Conserved Arl1 binding surface on the BIG1 DCB domain, Related to Figure 3.

(A) Surface representation of the BIG1 DCB domain colored according to evolutionary conservation. A UniProt search with 35% identity threshold produced 167 sequences as unique BIG1 orthologs, and these were used for ConSurf analysis. The Arl1 interaction surface is indicated in the inset images of the complex with $Arl1^{GTP}$ shown as ribbons.

(B) Alignment of the N-terminal region of all five proteins from humans that have a DCB domain: BIG1, BIG2, BIG3, MON2 and GBF1. Conserved residues are colored according to BLOSSUM62 with a 15% conservation threshold for visibility. Conserved residues specific to the BIG family (red dot above) or the GBF family (blue dot below) are indicated along with key residues in the $Arl1^{GTP}/DCB^{BIG1}$ interface (red bold) and the DCB-DCB interface (black bold), and the secondary structure of the DCB^{BIG1} domain.

(C) Sequence alignment of Arl1 orthologs, created as in (B). Switch 1 (red), interswitch (green, β-sheets depicted as arrows), switch 2 (orange), and key residues in the $Arl1^{GTP}/DCB^{BIG1}$ interface (arrowheads) are indicated.

Figure S4. Characterization of DCB^{BIG1} mutants, Related to Figure 4.

(A) Analysis of the thermal stability of DCB^{BIG1} mutants. The fraction of unfolded protein was determined using circular dichroism (CD). CD melting data were transformed using the fitted baselines from spectral data.

(B) Binding analysis of DCB^{BIG1} mutants. Ni-NTA beads loaded with His$_6$-DCB^{BIG1} variants were mixed with GST-Arl1$^{\Delta N14\text{-}GTP}$. Bound material was analyzed by SDS-PAGE and Coomassie staining.

(C) Determination of the dissociation constant of the Arl1$^{GTP/}$ DCB^{BIG1} complex by fluorescence anisotropy. Arl1^{GTP} was labeled with NT-495 and mixed with wild type DCB^{BIG1} or the indicated double mutants. Fitting to a 1:1 binding model is shown by a solid curve.

(D) Immunoblots from transiently transfected HeLa cells expressing wild-type or mutant versions of the HA-BIG1$^{1\text{-}559}$ construct, and probed for the HA epitope tag or for actin as a loading control.

Figure S5. Binding surfaces in Arl1-effector complexes, Related to Figure 6.

(A) Two views of the Arl1^{GTP}/DCB$^{\Delta 51\text{-}71}$ complex. α-helices 1, 4, 6, 8 from the DCB domain are depicted as ribbon diagrams. The key residues involved in the interaction are shown as sticks. The Arl1 switch region is shown as a surface representation with hydrophobic residues in yellow, polar residues in purple and charged residues in brown. GTP is shown in sticks and the Mg^{2+} ion as a sphere of green dots.

(B) Two views of the Arl1^{GTP}/GRIP^{Golgin245} complex with the GRIP domain as a ribbon diagram, coloring as in (A).

(C) Two views of the Arl1^{GTP}/BAR^{Arfaptin2} complex colored as in (A).

The Ile49 carbonyl group (C' I49) is highlighted in red in the three complexes, and the hydrophobic pocket is indicated with a red dashed circle.

Table S1 Data Collection and Refinement Statistics, Related to Figure 3

			$DCB^{\Delta 51\text{-}71}$-$Arl1^{GTP}$
Data collection			
	Space group		C121
	Unit cell dimensions		a=84.17 Å, b=50.73 Å, c=103.8 Å<br>α=90°, β=112°, Y=90°
	Resolution (Å)		29.05-2.28
	Rmerge		0.048 (0.667)
	I/σI		12.4 (1.7)
	Completeness (%)		97.3 (94.1)
	Redundancy		3.2 (3.2)
Refinement			
	Resolution (Å)		29.0-2.28
	No. of reflections		35386
	Rwork/Rfree		0.2589/0.2170
	No. of atoms		3008
		Protein	2895
		Ligand/ion	73
		Water	40
	B-factors		
		Protein	67.6
		Ligand/ion	63.7
		Water	59.6
	Rms deviation		
		Bond length (Å)	0.01
		Bond angles (o)	1.158
PDB ID			5EE5

Values in parentheses are for highest-resolution shell
